# Evaluation of the Outcomes of Patients Undergoing Colposcopy for High-Risk Human Papillomavirus Positivity and/or Abnormal Cervical Cytology

**DOI:** 10.3390/diagnostics16030465

**Published:** 2026-02-02

**Authors:** Necim Yalcin, Aysun Alci, Mustafa Gokkaya, Mehmet Goksu, Tayfun Toptas, Isin Ureyen

**Affiliations:** Department of Gynecologic Oncology, Saglik Bilimleri University Antalya Training and Research Hospital, Kazim Karabekir Street, 1, Antalya 07100, Turkey; aysun_alci@hotmail.com (A.A.); mugokkaya@gmail.com (M.G.); goksu0784@gmail.com (M.G.); drttoptas@gmail.com (T.T.);

**Keywords:** cervical cancer, cervical screening, cervical precancerous lesions, human papillomavirus, colposcopy

## Abstract

**Objectives:** The main objective of the present study was to evaluate the outcomes of patients referred for colposcopy due to human papillomavirus (HPV) positivity and/or abnormal cytology. **Methods:** A retrospective analysis was conducted on women who underwent colposcopy between January 2015 and December 2023. Demographic data and results of the colposcopy result were obtained from the patient files and the electronic gynecologic oncology clinic database. **Results:** A total of 2682 patients were included in the analysis. A cervical biopsy identified a cervical intraepithelial neoplasia (CIN)2+ (CIN2, CIN3, and invasive cancer) lesions in 361 patients (13.5%), while endocervical curettage (ECC) identified a CIN2+ lesions in 148 patients (5.6%). A total of 74 patients exhibited CIN2+ lesions in both cervical biopsy and ECC samples, while 74 patients displayed CIN2+ lesions exclusively in ECC samples. The distribution of high-risk HPV positivity in 435 patients with CIN2+ lesions revealed that 47.5% of patients were positive for HPV type 16, while 8.9% were positive for HPV type 18. A total of 50% of all patients diagnosed with CIN2+ lesions by ECC alone were found to be positive for HPV type 16 (37/74). Of the 116 patients with high-risk HPV positivity and normal cytology, 34 (29.3%) were high-risk HPV other-positive. **Conclusion:** HPV type 16 and 18 positivity represents the highest-risk groups in terms of CIN2+ lesion development. ECC should be considered, in particular, in women with HPV 16 positivity. Colposcopy should be performed immediately, rather than after one year, in women with high-risk HPV other-positivity and normal cervical cytology, in order to increase the detection rate of CIN2+ lesions.

## 1. Introduction

According to GLOBOCAN data, cervical cancer is the fourth most common cancer in women worldwide with approximately 660,000 new cases and 350,000 deaths in 2022. Approximately 94% of these 350,000 cervical cancer deaths occurred in low- and middle-income countries [1]. Almost all cases of cervical cancer are due to persistent infection with high-risk human papillomavirus (hr HPV) [2]. The development of cervical cancer is preceded by a long period of marked cervical cytological and histological abnormalities [3]. Cervical cancer can be prevented through early detection, as it has precursor abnormalities that can be successfully detected and treated through screening procedures using relatively simple technologies. Implementation of well-structured screening programmes that cover the entire target population and ensure the detection of cervical cytological and histological abnormalities followed by meticulous monitoring of treatment and follow-up of women has the potential to reduce the incidence of cervical cancer by up to 80% [4].

A cervical cancer screening programme consists of a number of stages. A community-based screening programme includes identification of a specific target age group, referral of women at high risk of precancerous lesions for colposcopy and biopsy, and treatment of women with confirmed precancerous lesions. Currently, there are three main alternatives for cervical cancer screening: cytology, HPV testing and cytology-HPV co-testing [5].

In 2020, the World Health Organization (WHO) initiated the Cervical Cancer Elimination Program with the objective of eliminating cervical cancer as a public health concern by 2030. If the programme is realised, by 2030, 90% of 15-year-old girls worldwide will have received the HPV vaccine, 70% of women aged 35–45 will have been screened with a high-sensitivity test, and 90% of women diagnosed with cervical cancer will have access to treatment and care. Consequently, the incidence will have fallen to a rate of four cases per 100,000 woman-years, which is the threshold for elimination as a public health problem [6].

A population-based cervical cancer screening programme utilising conventional cytology was first initiated in Turkey in 2004. In light of the growing prevalence of HPV-based tests, the Turkish Ministry of Health issued a cervical cancer screening guideline in 2014, which recommends the use of both HPV and conventional cytology (one visit, double triage strategy) in conjunction with one another. In accordance with this guideline, women between the ages of 30 and 65 were screened for cervical cancer every five years. Patients with HPV 16 and/or 18 positivity or hr HPV other-positivity and abnormal cytology were referred for colposcopy [7].

The present study aimed to evaluate the outcomes of patients referred to a tertiary healthcare institution for colposcopy due to hr HPV positivity and/or abnormal cytology.

## 2. Material and Method

The present study retrospectively examined a cohort of women who underwent colposcopy between 1 January 2015 and 31 December 2023. A colposcopic examination is indicated in cases where there are abnormal cytological results (Low-grade intraepithelial lesion (LSIL), Atypical squamous cells cannot exclude high-grade squamous intraepithelial lesion (ASC-H), High-grade squamous intraepithelial lesion (HSIL), Atypical glandular cell (AGC)), recurrent Atypical Squamous Cells of Undetermined Significance (ASC-US), hr HPV positivity (including patients with HPV types other than 16 and 18, with normal cytology) and suspicious for carcinoma. The Hybrid Capture 2 (Qiagen, Hilden, Germany) test was used for HPV testing. In instances where HPV positivity was detected using the Hybrid Capture 2 (Qiagen) test, genotyping was performed using the CLART kit (Genomica, Madrid, Spain). Patients who had undergone hysterectomy and patients diagnosed with gynecological cancer were excluded from the study. The data set encompasses variables such as age, menopausal status, smoking status, and pathological results of colposcopy-guided biopsies and endocervical curettage (ECC). These data were retrieved from patient files and the institution’s electronic database system. The study was initiated subsequent to ethical committee approval from the Health Sciences University Antalya Training and Research Hospital Ethics Committee on 28 December 2023, under number 2023-330. The study was conducted in accordance with the ethical principles outlined in the Declaration of Helsinki. The colposcopy examination database was evaluated to ascertain the appropriateness of colposcopy procedures. It is noteworthy that all colposcopies were performed by gynecologic oncologists within the specified time period. Colposcopic evaluation was performed subsequent to the application of a 3% acetic acid solution to the cervix. Cervical biopsies were performed in cases where lesions indicative of cervical intraepithelial neoplasia (CIN) were observed during colposcopic examination. In instances where no atypical colposcopy findings were detected, random biopsies were obtained at the discretion of the physician conducting the colposcopy. ECC was performed when colposcopy was inadequate, for example, when the cervix was obscured by haemorrhage, inflammation or scarring, when the squamocolumnar junction (SCJ) was not visible or only partially visible, when the transformation zone (TZ) was type 3, when the visualised lesion extended into the endocervical canal, or in the presence of HSIL and ASC-H cytological results. In the absence of the above factors, ECC was sometimes performed according to the clinical judgement of the colposcopist. The ECC was performed using a Novak curette to excavate the endocervical canal. The tissue was then prepared as a histopathological specimen.

The pathological results of cervical or ECC biopsies performed at our centre were evaluated in two categories based on the treatment threshold: cases below CIN2 level and cases at or above CIN2 level. Cases categorised at or above CIN2 level included CIN2, CIN3, and invasive cancer.

### Statistical Analysis

The data were analysed using SPSS version 22.0. Descriptive statistics were employed. The binary variables were expressed as frequencies and percentages. A Chi-square test was employed for the purpose of comparing proportions across different groups.

## 3. Results

A total record of 2699 patients were obtained from the institution’s electronic records. A total of 17 patients were excluded from the study due to the absence of a uterus and cervix as a result of surgical intervention. The total number of patients included in the analysis was 2682. Analyses revealed that the median age of the cohort was 44 years (range, 21–79 years). Among the patients for whom data on their smoking status was available, 28.6% were identified as current smokers. 69% of cases with known menopausal status were premenopausal. The presentation of the detailed cytology results is outlined in Table 1. Following the exclusion of patients without HPV results and those with negative HPV, the rates of patients with HPV 16, HPV 18, HPV 16 + 18 and hr HPV other-positivity were 34.7%, 8.9%, 3.3% and 53.1%, respectively.

The colposcopic findings were reported as normal in 1083 patients (40.4%), inadequate in 50 patients (1.9%), and abnormal in 1549 patients (57.8%). Of the 2682 patients included in the study, 435 (16.2%) exhibited evidence of CIN2+ lesions based on cervical biopsy and ECC results. A cervical biopsy identified a CIN2+ lesion in 361 patients (13.5%), while ECC identified a CIN2+ lesion in 148 patients (5.6%) (Table 2). A total of 74 patients exhibited CIN2+ lesions in both cervical biopsy and ECC samples, while 74 patients displayed CIN2+ lesions exclusively in ECC samples. Of the 435 patients with CIN2+ lesions on colposcopic biopsy, 330 (75.8%) underwent conization. The primary reasons for not performing conization on patients with CIN2+ lesions are that they wish to continue their treatment at another centre and are concerned about future pregnancies. The distribution of cervical biopsy and ECC results among the whole group is presented in Table 2.

A review of the distribution of hr HPV positivity in 435 patients with CIN2+ lesions revealed that 47.5% of patients were positive for HPV type 16, while 8.9% were positive for HPV type 18. Of the 207 patients with HPV type 16 positivity and CIN2+ lesions, 170 were diagnosed by cervical biopsy, 40 by both cervical biopsy and ECC, and 37 by ECC only. Similarly, of the 39 patients with HPV type 18 positivity and CIN2+ lesions, 34 were diagnosed by cervical biopsy, 5 by both cervical biopsy and ECC, and 5 by ECC only.

In the cohort of patients with HPV 16 positivity, cervical biopsy revealed CIN2+ lesions in 20.2% (*n* = 170/839) of patients. In comparison, the prevalence of CIN2+ lesions was 15.6% (*n* = 34/218), 19.5% (*n* = 15/77) and 8.9% (*n* = 111/1243) in patients with positivity for HPV 18, HPV 16 + 18 and hr HPV other, respectively. The distribution of cervical biopsy results with regard to HPV status is presented in Table 3.

In the cohort of patients with HPV 16 positivity, ECC revealed CIN2+ lesions in 9.2% (*n* = 77/839) of patients, while the corresponding figure was 4.6% (*n* = 10/218), 6.5% (*n* = 5/77) and 3.2% (*n* = 40/1243) in patients with positivity of HPV 18, HPV 16 + 18 and HPV other, respectively (Table 4). A total of 77 patients, representing 52% of the 148 patients, were found to be positive for HPV type 16 when we examined patients with CIN2+ lesions detected with ECC. The distribution of ECC results with regard to HPV status is presented in Table 3.

If the patients who did not undergo ECC were excluded, the rates were 11.4% (*n* = 77/672), 5% (*n* = 10/198), 7.2% (*n* = 5/69) and 4.1% (*n* = 40/967), respectively, for patients with positivity of HPV 16, HPV 18, HPV 16 + 18 and HPV other.

In the cohort of patients without cervical biopsy and in those whose cervical biopsy revealed normal cervical tissue, 1797 patients had ECC, and 4.1% (*n* = 74/1797) of these patients had CIN2+ lesions in their ECC. This equates to 2.7% of the total number of patients included in the study. Among the 1797 patients, those with HPV 16 positivity demonstrated a 6.9% (37/536) prevalence of CIN2+ lesions based on ECC results. The corresponding rates were 2%, 3.6%, and 3.1% in the groups with HPV 18, HPV 16 + 18, and HPV other-positivity, respectively. A total of 50% of all patients diagnosed with CIN2+ lesions by ECC alone were found to be positive for HPV 16 (37/74). The data regarding the cervical biopsy and ECC results in the group of patients with HPV positivity and normal cytology were shown in Table 4.

In the cohort of patients with hr HPV positivity and normal cervical cytology, cervical CIN2+ lesions were identified by cervical biopsy in 116 patients and by ECC in 44 patients, representing 4.3% and 1.6% of the study population, respectively. Among patients with CIN2+ lesions identified via cervical biopsy, the prevalence of CIN2+ lesions were 16.3% in the HPV 16-positive group, 8.3% in the HPV 18-positive group, and 5.9% in the hr HPV other-positive group. Of the 116 patients with hr HPV positivity and normal cytology, 34 (29.3%) were hr HPV other-positive.

## 4. Discussion

In the present study, 2682 patients who were referred to a tertiary healthcare institution and were eligible for colposcopy were evaluated. A total of 423 patients (16.2%) were diagnosed with CIN2+ lesions, and 75.8% of these patients underwent conization.

A review of the HPV subtypes in patients referred to colposcopy due to hr HPV positivity revealed that the majority of HPV subtypes were hr HPV other-positivity. In present study, HPV 16 positivity was found to be 34.7% and hr HPV other-positivity rate was 53.1%. Upon examination of existing literature, similar results were observed in the present study. It was reported that the majority of patients referred to colposcopy were hr HPV other-positivity, with a rate varying between 59.5% and 76.4% [8,9].

In the present study, CIN2+ lesions were detected in 16.2% of 2682 patients, with 75.8% of these patients undergoing conization. A review of the literature reveals numerous studies evaluating patients who underwent colposcopy due to hr HPV positivity and/or abnormal cervical cytology. These studies concur with the findings of the present study, which found that the rate of detection of CIN2+ lesions after colposcopy ranged from 7.5% to 31.1% [7,10,11,12,13,14,15,16].

It is believed that infection with oncogenic HPV types plays a role in the development of almost all cervical cancers and precancerous lesions. HPV types 16 and 18 are thought to be responsible for approximately 70% of all cervical cancers. In the present study, 47.5% of 435 patients with CIN2+ lesions after colposcopic biopsy were found to be HPV type 16 positive, while 8.9% were HPV type 18 positive. A review of similar studies revealed that HPV type 16 and HPV type 18 were detected most frequently in high-grade lesions and invasive cancers as a result of colposcopic biopsy. This rate varied between 48.2 and 69% [17,18,19,20,21,22].

The colposcopy examination is limited in its ability to evaluate the endocervical canal; therefore, ECC procedure has traditionally been used for this purpose. In 2006, the American Society for Colposcopy and Cervical Pathology (ASCCP) stated that ECC would be the preferred method for patients with abnormal cervical cancer screening results where no lesions are seen on colposcopy and the squamocolumnar junction (SCJ) is not completely visible. However, the ASCCP also stated that ECC is acceptable in all cases. However, in the recent risk-based colposcopy standards guideline published in 2017, the ASCCP standards for ECC were postponed [23,24].

In the present study, 148 patients who underwent ECC exhibited CIN2+ lesions, representing 5.5% of the entire study population. In patients without cervical biopsy or with normal cervical tissue on cervical biopsy, a further 74 patients were diagnosed with CIN2+ lesions by ECC alone. In the literature, the rate of CIN2+ lesion detection with ECC varies between 4.2% and 14.4%. This appears to be consistent with the findings of the present study [25,26,27,28,29]. A review of the literature also reveals that the rate of CIN2+ lesion detection with ECC alone varies between 0.5% and 6.4%. This rate appears to be consistent with the present study, which reports a rate of 2.7% [27,28,29,30,31]. The prevalence of HPV type 16 positivity was 52% among patients with CIN2+ lesions detected by ECC. None of the HPV-negative patients had HSIL detected by ECC, and only one HPV-negative patient had invasive cancer. Concurrently, HPV type 16-positive patients accounted for 50% of all patients diagnosed with CIN2+ lesions by ECC alone (37/74). This indicates that HPV type 16 positivity is associated with an increased likelihood of detecting CIN2+ lesions by ECC, as observed in the study by Liu, A. H. C. et al. (24.4% vs. 11.5% in women with and without HPV 16 infection, *p* < 0.05). Consequently, endocervical sampling with ECC may be recommended, particularly in patients with HPV type 16 positivity [25].

The majority of women with hr HPV positivity during cervical cancer screening will have normal cervical cytology. According to the ASCCP guidelines, HPV type 16 and 18 positivity with normal cervical cytology is an indication for colposcopy, while co-testing after one year is recommended for patients with hr HPV other-positivity if cervical cytology results are normal [32]. In the present study, cervical biopsy revealed CIN2+ lesions in 116 (10%) patients who had undergone colposcopy due to hr HPV positivity and normal cytology and ECC revealed CIN2+ lesions in 44 (3.8%) patients. In patients with normal cytology, 58.6% of patients with CIN2+ lesions on cervical biopsy and 59% of patients with CIN2+ lesions on ECC were found to be HPV type 16 positive. In the same group of patients, 29.3% of those with CIN2+ lesions detected by cervical biopsy and 27.2% of those with CIN2+ lesions detected by ECC were also positive for hr HPV other. In accordance with the present study, Zhang J. et al. and Wang Z. et al. recently found that 7.7% and 20.4% of patients who underwent colposcopy due to hr HPV positivity and normal cervical cytology, respectively, had CIN2+ lesions. As in the other two studies, the majority of HPV-positive and normal cytological cases in this study were found to be HPV 16 positive [33,34].

Although the management of HPV type 16 and 18 positivity and normal cytology is well established, there is controversy in the management of hr HPV other-positivity and normal cytology. The follow-up of these women according to current guidelines poses a significant difficulty due to low compliance and discontinuation rates. Additionally, several studies have shown that certain strains of hr HPV other may exhibit a comparable or even higher risk of developing HSIL lesions than HPV type 18 [35,36]. Furthermore, it has been documented that the incidence of false negative results in cervical cytology tests ranges from 15 to 65% [37]. This situation raises a number of questions regarding the follow-up of non-invasive hr HPV other positive cases a year later, given the possibility of an increased risk of cervical dysplasia.

Within the present study, CIN2+ lesions were observed in 5.9% of patients with hr HPV other-positivity and normal cervical cytology on cervical biopsy. This rate was found to be higher than that reported in previous studies [32,38]. We believe that the most important reason for this is that all colposcopic procedures are performed by experienced colposcopists, and because of our role as a tertiary referral centre, extra random cervical biopsies are taken with the aim of detecting any existing lesions.

In the present study, CIN2+ lesions were found in 8.3% of women with HPV 18 positivity and normal cervical cytology and in 5.9% of women with hr HPV other-positive and normal cervical cytology, and contrary to the studies referenced above, HPV 18 positivity has a higher rate of CIN2+ lesions than hr HPV other.

Among patients with hr HPV positivity and normal cervical cytology whose colposcopy revealed CIN2+ results, 29.3% (*n* = 34/116) of patients were hr HPV other-positive. It is postulated that performing colposcopy instead of follow-up in hr HPV other-positivity will result in an increased number of cervical precancerous and cancer diagnoses, as well as an increased financial burden and a negative impact on patient-based outcomes due to the additional colposcopy requirements.

The study has several advantages, including a large number of patients, being a tertiary referral centre and all colposcopy procedures being performed by an experienced gynaecological oncology team. However, the study also has several disadvantages. These include a retrospective study design, lack of access to some demographic characteristics of patients such as smoking and parity, lack of cytology results in some patients, and cytology results that were not subjected to statistical analysis because the majority of cytology results were LSIL and lower than LSIL. Additionally, due to the retrospective design of the study, the diagnostic algorithm could not be fully standardized. In particular, the lack of systematic colposcopic biopsy in all HPV-positive but cytology-negative patients may have led to the overlooking of some CIN2+ lesions and could have affected the diagnostic sensitivity of the study.

## 5. Conclusions

It can be stated that HPV type 16 and 18 positivity represents the riskiest groups in terms of CIN2+ lesion development. It is therefore recommended that ECC should be considered, in particular in women with HPV 16 positivity. Furthermore, it is proposed that colposcopy should be performed immediately, rather than after one year, in women with hr HPV other-positivity and normal cervical cytology, in order to increase the detection rates of CIN2+ lesions.

## Figures and Tables

**Table 1:** The distribution of cytology results of the whole cohort.

	Frequency	Percent (%)
NILM	810	30.20
Infection	412	15.36
Inadequate	306	11.41
ASCUS	197	7.34
LSIL	314	11.71
ASC-H	53	1.98
HSIL	64	2.39
AGC	31	1.16
Not known *	494	18.41
AIS	1	0.04
**Total**	2682	100

* These patients admitted with human papillomavirus positivity without cytology result. Abbreviations: NILM: Negative for intraepithelial lesion or malignancy, ASCUS: Atypical Squamous Cells of Undetermined Significance, LSIL: Low-grade intraepithelial lesion, ASC-H: Atypical squamous cells cannot exclude high-grade squamous intraepithelial lesion, HSIL: High-grade squamous intraepithelial lesion, AGC: Atypical glandular cell, AIS: Adenocarcinoma in situ.

**Table 2:** The distribution of cervical biopsy and ECC results among whole group.

Cervical Biopsy	Frequency	Percent (%)	ECC	Frequency	Percent (%)
Not performed	1089	40.6	Not performed	583	21.7
<CIN2	1232	45.9	<CIN2	1951	72.7
CIN2 + CIN3	343	12.8	CIN2 + CIN3	141	5.3
Invasive cancer	18	0.7	Invasive cancer	7	0.3
**Total**	2682	100	**Total**	2682	100

Abbreviations: ECC: Endocervical curettage, CIN: Cervical intraepithelial neoplasia.

**Table 3:** The distribution of cervical biopsy and ECC results with regard to HPV status.

HPV Status		Cervical Biopsy				Total
		Not Performed	Normal	HSIL	Invasive Cancer	
	Not known	78 (41.7%)	81 (43.3%)	26 (13.9%)	2 (1.1%)	187 (100%)
	Negative	20 (50%)	17 (42.5%)	2 (5%)	1 (2.5%)	40 (100%)
	HPV 16 ^1^	298 (35.5%)	371 (44.2%)	161 (19.2%)	9 (1.1%)	839 (100%)
	HPV 18 ^2^	83 (38%)	101 (46.5%)	32 (14.6%)	2 (0.9%)	218 (100%)
	HPV 16 + 18 ^3^	14 (18.2%)	48 (62.3%)	14 (18.2%)	1 (1.3%)	77 (100%)
	HPV other ^4^	518 (41.7%)	614 (49.4%)	108 (8.7%)	3 (0.2%)	1243 (100%)
**HPV Status**		**ECC**				**Total**
		**Not Performed**	**Normal**	**HSIL**	**Invasive** **Cancer**	
	Not known	23 (12.3%)	149 (79.7%)	15 (8%)	0 (0%)	187 (100%)
	Negative	11 (27.5%)	28 (70%)	0 (0%)	1 (2.5%)	40 (100%)
	HPV 16 ^1^	167 (19.9%)	595 (70.9%)	73 (8.7%)	4 (0.5%)	839 (100%)
	HPV 18 ^2^	20 (9.2%)	188 (86.2%)	9 (4.1%)	1 (0.5%)	218 (100%)
	HPV 16 + 18 ^3^	8 (10.4%)	64 (83.1%)	5 (6.5%)	0 (0%)	77 (100%)
	HPV other ^4^	276 (22.2%)	927 (74.6%)	39 (3.1%)	1 (0.08%)	1243 (100%)

^1^ includes patients with only HPV 16 and the patients who had both HPV 16 and types other than HPV 16 and HPV 18; ^2^ includes patients with only HPV 18 and the patients who had both HPV 18 and types other than HPV 16 and HPV 18; ^3^ includes patients with HPV 18, HPV 16 and other high risk HPV types; ^4^ includes the patients who had HPV types other than HPV 16 and HPV 18. Abbreviations: ECC: Endocervical curettage, HPV: Human papillomavirus, HSIL: High-grade squamous intraepithelial lesion.

**Table 4:** The distribution of cervical biopsy and ECC results among patients with HPV positivity and normal cytology.

		Cervical Biopsy				Total	ECC				Total
		Not Performed	Normal	HSIL	Invasive Cancer		Not Performed	Normal	HSIL	Invasive Cancer	
**HPV**	HPV 16	192 (45.9%)	158 (37.8%)	66 (15.8%)	2 (0.5%)	418 (100%)	108 (25.8%)	284 (67.9%)	25 (6.0%)	1 (0.2%)	418 (100%)
	HPV 18	58 (48.3%)	52 (43.3%)	10 (8.3%)	0 (0.0%)	120 (100%)	13 (10.8%)	102 (85.0%)	5 (4.2%)	0 (0.0%)	120 (100%)
	HPV 16 + HPV 18	9 (25%)	23 (63.9%)	4 (11.1%)	0 (0.0%)	36 (100%)	4 (11.1%)	31 (86.1%)	1 (2.8%)	0 (0.0%)	36 (100%)
	HPV-other	287 (49.6%)	258 (44.6%)	34 (5.9%)	0 (0.0%)	579 (100%)	197 (34.0%)	370 (63.9%)	12 (2.1%)	0 (0.0%)	579 (100%)
**Total**		546 (47.3%)	491 (42.6%)	114 (9.9%)	2 (0.2%)	1153 (100%)	322 (27.9%)	787 (68.3%)	43 (3.7%)	1 (0.09)	1153 (100%)

Abbreviations: ECC: Endocervical curettage, HPV: Human papillomavirus, HSIL: High-grade squamous intraepithelial lesion.

## Data Availability

The raw data supporting the conclusions of this article will be made available by the authors on request.
